# Global progress against cancer—challenges and opportunities

**DOI:** 10.7497/j.issn.2095-3941.2013.04.001

**Published:** 2013-12

**Authors:** Frédéric Biemar, Margaret Foti

**Affiliations:** American Association for Cancer Research, Philadelphia, PA 19130, USA

**Keywords:** Collaboration, prevention, translational research, genetics, genomics, epigenetics, immunotherapy

## Abstract

The last ten years have seen remarkable progress in cancer research. However, despite significant breakthroughs in the understanding, prevention, and treatment of cancer, the disease continues to affect millions of people worldwide. Cancer’s complexity compounded with financial, policy and regulatory roadblocks has slowed the rate of progress being made against cancer. In this paper, we review a few of the most recent breakthroughs that are fueling medical advances and bringing new hope for patients affected by this devastating disease. We also address the challenges facing us and the opportunities to accelerate future progress against cancer. The efforts of the American Association for Cancer Research (AACR) to address the cancer burden already extend beyond the borders of the United States of America. The AACR is committed to increasing its efforts to stem the tide of cancer worldwide by promoting innovative programs, strategies, and initiatives for cancer researchers and all those engaged in cancer-related biomedical sciences around the world.

## Introduction

The past four decades have seen significant progress in the understanding, diagnosis, treatment, and prevention of cancer. However, as cancer mortality rates have declined in the U.S. and other developed countries, cancer incidence and mortality continue to rise around the world. The latest World Health Organization (WHO) statistics predicted 13.2 million cancer-related deaths worldwide by 2030, which is up from 7.6 million in 2008 when the last report was published. With the continuing growth and aging of the world’s population, the global burden of new cancer cases is estimated to rise from 12.7 million in 2008 to 20.3 million by 2030^1^^,^^2^.

The causes of the predicted increase in the global cancer burden are numerous and relatively well established. In addition to aging, these include: lifestyle risk factors such as tobacco use, diet and obesity, lack of exercise, alcohol consumption, and excessive exposure to sunlight; environmental and occupational exposures to carcinogens and mutagens (including chemicals and radiation); infectious agents (*Helicobacter pylori*, hepatitis B virus, hepatitis C virus, human papillomavirus, Epstein-Barr virus); chronic inflammation; hormone metabolism; family history; and ethnicity and socioeconomic status.

The American Association for Cancer Research (AACR) believes that the conquest of cancer, through further scientific progress in cancer etiology, prevention, diagnosis, treatment, and quality healthcare delivery, must become an international priority. There is no doubt that the looming global cancer crisis requires global solutions. It is our conviction that, by working collaboratively with governments around the world, relevant institutions, and key individual stakeholders in the cancer field, we have the potential to develop innovative approaches that will be effective in expediting advances against cancer and save more lives from this disease.

## Progress against cancer

The realization that gene damage and genetic mutations can cause cancer was arguably one of the greatest research advances in the modern era, one that paved the way for therapies that target specific cancer-driving defects. During the 1970s, scientists identified two families of genes—proto-oncogenes and tumor suppressor genes—that normally regulate the natural processes of cell growth and death in healthy tissues and organs. Oncogenes, the mutated form of proto-oncogenes, encode faulty proteins involved in the genesis and spread of tumors. Tumor suppressor genes typically control molecular programs that negatively regulate cell proliferation. When their gatekeeper role is altered by mutations, cell growth and proliferation goes awry. Further research subsequently established that tumors are more than insular masses of proliferating cancer cells. It also quickly became clear that the contributions of the tumor microenvironment greatly influenced tumorigenesis. Therefore, tumors should be viewed as complex tissues composed of multiple distinct cell types interacting with one another. In 2000, Hanahan and Weinberg proposed the concept of cancer “hallmarks”: six essential capabilities that normal cells acquire as they evolve progressively to a neoplastic state^3^. This conceptual framework, which was largely vindicated by subsequent discoveries over the last decade, was recently revisited and expanded^4^.

In this paper, we review a few of the most recent breakthroughs that are fueling medical advances and bringing new hope for patients affected by this devastating disease.

The last ten years have seen remarkable progress in cancer research, aided by technological advances such as the so-called “next-generation sequencing” (NGS). Thanks to large-scale collaborative efforts such as the “International Cancer Genomics Consortium” (ICGC), as well as other initiatives, we now have an ever-increasing understanding of the genes and molecular pathways that are disrupted in cancer. This knowledge has yielded a cornucopia of new therapies based on drugs that precisely target these alterations. Molecular targeted therapies are now an integral component of cancer patient care, alongside the three pillars of cancer treatment that are surgery, radiation, and chemotherapy. Cancer genomics, while revealing a treasure trove of information about the basic biological processes that become perturbed in cancer, has also made it abundantly clear that cancer is not one disease. Tumors are extremely heterogeneous, with more than 200 different types and subtypes currently recognized^5^^,^^6^. This has triggered a paradigm shift in the standard of care, away from a one-size-fits-all approach and toward individual or “tailored therapy” whereby treatment decisions are based on the mutational landscape of a patient’s tumor. This approach, commonly referred to as personalized cancer medicine or precision medicine, is still very much in early development, but it clearly represents the future of cancer care.

Other examples of important technological advances include the successful application of nanotechnology in cancer biology, which has spurred the development of new agents and targeted delivery systems that bring new hope for treatments that are not only more efficient, but also much less toxic. One of the most recent success stories include the highly encouraging results obtained with the nanodrug form of paclitaxel in the treatment of non-small cell lung cancer and also metastatic pancreatic cancer^7^^,^^8^.

Among the promising areas of basic cancer research, epigenetics has recently moved to center stage. Most cancer cells have profound abnormalities in their epigenomes when compared to normal cells of the same tissue. Unlike genetic mutations, however, epigenetic changes are potentially reversible. This has led to renewed interest for existing epigenetic drugs and the development of new ones. Researchers have begun to explore avenues to integrate epigenetic therapy into patient care, and early success is already apparent in the case of lymphoma, leukemia, and lung cancer^9^^,^^10^.

Another exciting field of research involves immunotherapy as a treatment for cancer which, after many decades of slow progress, is a concept whose time has finally arrived. In the past three years, various cancer immunotherapies including cancer vaccines, adoptive cell transfer, and checkpoint blockade have shown remarkable and durable responses in patients in a number of cancer types. These include: metastatic melanoma, renal cell carcinoma, non-small cell lung cancer, prostate cancer, colorectal cancer, pancreatic cancer, and leukemia and lymphoma^11^.

Cancer prevention research is another area that has benefited from important technological advances. Improved imaging capabilities and newly developed non-invasive imaging agents are now available that allow more precise cancer diagnoses and inform treatment interventions. For instance, ultrasound imaging has greatly improved breast cancer detection by allowing the identification of small tumors in dense breasts. Novel technologies to detect, isolate, and analyze circulating tumor cells have recently been developed that help characterize a patient’s tumor without surgery and inform decision on the best therapeutic approach.

## Challenges and opportunities

Decades of research have provided insights into the numerous factors that are germane to cancer and have shown that the majority of cancers diagnosed today are the result of preventable causes. This knowledge has been translated into new clinical approaches, as well as public education and policy initiatives. However, despite the demonstrated success of public health campaigns, convincing individuals about the importance of behavioral and lifestyle changes remains an upward battle. Nevertheless, cancer prevention and early detection continue to represent areas where effective strategies have the potential to yield the greatest impact on overall reduction of morbidity and mortality from this disease. Global cancer surveillance and cancer control programs must be strengthened with appropriate resources. When population-based screening programs have been implemented, they have almost invariably been met with a dramatic increase in 5-year survival for the cancers they detect. Continued research to develop new and better methods for the identification of high-risk individuals is paramount if we are to reduce the burden of a disease that is largely preventable. Risk stratification will also help address the legitimate concerns that heightened surveillance may lead to overdiagnosis and overtreatment.

Another challenge is the significant gap that persists between basic research and clinical application, despite the clear progress over the past 5 years. The enormous potential of translational cancer research will only be realized when impediments are systematically addressed. More precise diagnoses and treatments will require improved genomics-based technologies, large repositories and interactive data networks, and better mathematical and computer models to analyze and exploit the deluge of “big data” generated by large-scale systematic cancer genomics projects such as those mentioned earlier. There is also a clear need for standardized and uniform policies and guidelines for the isolation, processing, analysis, and annotation of biospecimens. The ability to implement innovative clinical trial designs will require ongoing dialogue between basic scientists and clinical researchers. Drug development advances will necessitate animal models with better predictive value, as well as continued efforts in biomarker discovery. Despite evident success, rational drug development remains a complex, high-risk, and time-consuming process that might greatly benefit from coordinated pre-competitive collaborations among all sectors in the field; from academia to government to the biotechnology and pharmaceutical industry, but also philanthropic organizations and patient advocacy groups.

Finally, advances have not been uniform for all types of cancer, or for all patients with a certain type of cancer. In addition, the burden of cancer is not evenly distributed across populations. Thus, even in developed countries, cancer remains a significant problem despite the considerable progress mentioned above. Furthermore, in low- and middle-income countries (LMIC) that are transitioning to higher levels of development, reduction in infection-related cancers is unfortunately often accompanied by an increase in cancers related to a Westernization of lifestyle. Of all major causes of disease worldwide, cancer has the greatest economic burden from premature death and disability. Its global economic toll is 20% higher than that from any other major disease^12^. In this respect, the inclusion of cancer in the 2011 United Nations Declaration on Non-Communicable Diseases that has a commitment to reduce preventable deaths by 25% by the year 2025 is not only commendable, but also highly timely.

In conclusion, we have entered a new era of pioneering breakthroughs in cancer research. Scientific opportunities to markedly reduce the global cancer burden exist today, but more resources are needed to fully realize the potential of today’s science and technology. The AACR strongly believes that research is our best defense against cancer. Continued progress against a disease that is highly heterogeneous and deceptively adaptive will require a sustained, collaborative, and global effort. This includes investing in the talent, tools, and infrastructure to support cancer research and biomedical science.
